# Effects of non-Darcy mixed convection over a horizontal cone with different convective boundary conditions incorporating gyrotactic microorganisms on dispersion

**DOI:** 10.1038/s41598-022-18549-2

**Published:** 2022-10-04

**Authors:** M. Ferdows, Bader Alshuraiaan, Nayema Islam Nima

**Affiliations:** 1 Research Group of Fluid Flow Modeling and Simulation, Department of Applied Mathematics, University of Dhaka, Dhaka, 100 Bangladesh; 2 Department of Mechanical Engineering, Kuwait University, 13060 Safat, Kuwait; 3 Department of Quantitative Sciences, International University of Business Agriculture and Technology, Dhaka, 1230 Bangladesh

**Keywords:** Biophysics, Mathematics and computing, Physics

## Abstract

This paper investigates the influence of dispersion impact on mixed convection flow over a horizontal cone within a non-Darcy porous medium. Multiple convective boundary conditions are applied to address the heat, mass and motile microorganism transfer phenomena. This paper incorporates the dispersion effect for gyrotactic microorganisms due to biological and environmental applications. By imposing appropriate similarity transformations, the nonlinear partial differential equations governing flow, temperature, concentration, and microbe fields are reduced to a system of ordinary differential equations & then solved using the MATLAB BVP4C function. The computation of grid independence test is analyzed for different flow profiles to show the precision of the points. In a few instances, our present numerical data is compared with previously published works, leading to excellent agreement. The non-Darcy effect, as well as mixed convection values from 0.1 to 0.9 and buoyancy parameters from 0.2 to 0.8, all significantly affects the velocity profile. The reduction in the microorganism profile is brought on by the increase in the bioconvection Lewis parameter and bio convection peclet number between 0.3 and 1. In the absence of dispersion, the variation of Biot numbers between 0.5 and 2, favor heat, mass, and motile microorganism transfer the most in the range of mixed convection parameter 0.5 to pure forced convection 1. Thermal, solutal and microorganism dispersion coefficients a, b, c that lie between $\frac{1}{7}$ and $\frac{1}{3}$ and higher values of modified peclet number ranges from 2 to 10 cause increased dispersion effects which lower flow transfer rates mostly in forced convection regime.

## Introduction

Convection in a porous fluid-saturated media from an axisymmetric bodies(such as a cone, cylinders, spheres) have attracted numerous researchers as a result of its numerous engineering applications and geophysics such as thermal insulation, geophysical flows, the cooling of electronic systems, ground water hydrology, chemical catalytic reactor, ceramic process, petroleum reservoirs, ground water pollution, filtration process etc. Complete reviews of convective heat transport in porous media have been written by Nield and Bejan^1^, Pop and Ingham^2^ and Bejan^3^. In the last few decades, a number of studies on mixed convective heat and mass transfer have been published, employing both Darcian and non-Darcian porous media. Srinivasachary and Reddy^4,5^ discovered natural and mixed convection for power law fluid in Darcy porous media, while Lai^6^ used the Darcy model to observe mixed convection in porous media. Non-Darcian models are Darcy formulations that include inertial drag, vorticity diffusion, and combinations of these components, according to^7^. Various models for assessing non-Darcian flow in porous media, such as Brinkman-extended Darcy, Forchheimer-extended Darcy, and generalized stream models, have been published in the literature^7–11^. The non-Darcy model discussed in this study is a continuation of the classical Darcian formulation achieved by including a squared velocity factor to account for inertial effects in the momentum equation.

In all the above studies thermal dispersion effects are ignored. Many researchers^12–18^ have looked at the influence of thermal dispersion on convective heat transfer through porous media in a variety of situations. Plumb^12^ stated that when inertial effects are dominant, the thermal dispersion effects in a porous media become important. Effects of thermal dispersion on convection for non-Darcy porous media are studied by Amin^13^ and Kairi^14^. And the effects of thermal dispersion for mixed convection flow with nanofluid through vertical surfaces are observed in Refs.^16–18^. Heat and mass transfer analysis using a convective boundary condition is a significant and relevant consideration in the gas turbines, nuclear reactors, and heat exchangers industries. Heat is provided to the convecting fluid via a boundary surface with a finite heat capacity, which provides a convective heat transfer coefficient in this mechanism (namely, Biot number). Given the nature of these applications, Hady^19^ studied non-Darcy natural convection with convective boundary condition containing microorganisms, mixed convection flow through vertical surfaces were studied in Refs.^20–23^ in recent years and also with the presence of convective boundary conditions, Mahady^24^ observed mixed convection boundary layer flow via a horizontal circular cylinder.

Now a day’s researchers are paying more attention to the evolution of microorganisms in bioconvection. Bioconvection occurs when the macroscopic flow of fluid causes greater flexibility in swimming microorganisms. The self-driving mobile microorganisms desire to increase the volume of base fluid in the system by producing a bioconvective stream in one direction, according to Refs.^25–29^. Chemical or oxytactical properties, gyrotactic traits, and negative gravitational characteristics are used to classify motile microorganism A bottom-heavy microbe with gyrotaxis is the most frequent. Kuznetsov^30^ came up with the idea of putting motile microorganisms into nanofluid. The addition of motile microbes to the solution provides a number of benefits, including improved mass transfer, microscale mixing, and fluid stability. Referenes^31–38^ provide important works on the use of bioconvection in thermobioconvection, microbial augmentation, bioMicrosystems, biofuels, and other bioengineering systems. Among them mixed convection containing gyrotactic microorganism flow with convective boundary conditions were observed in Refs.^31–33^ and also mixed convection phenomena with gyrotactic microorganism over cone were observed by Khan et al.^34^, Saleem et al.^35^. Waqas^39^ observed Magneto-Burgers Nanofluid Stratified Flow with Swimming Motile Microorganisms and also Hussian^40^ studied MHD nanofluid flow with convective boundary conditions containing gyrotactic microorganism.

The objective of this article is to study free forced convection having motile gyrotactic microorganisms under the effects of dispersion past horizontal cone with convective boundary conditions which has not been studied yet. The main moto of this study are (1) to examine mixed convection through heat mass and motile density, (2) to analyze flow characteristics in the presence and absence of dispersion effects, (3) to address multiple convective boundary conditions and also analyze the transfer rates of heat, mass, and motile microorganisms, (4) to analyze the impact of different profile distributions for single mixed convection which encompasses the full mixed convection regime, from pure forced convection to pure free convection. (5) To compare present results with previous published results in order to validate the accuracy of the present model.

## Model formulation

**Figure 1:**
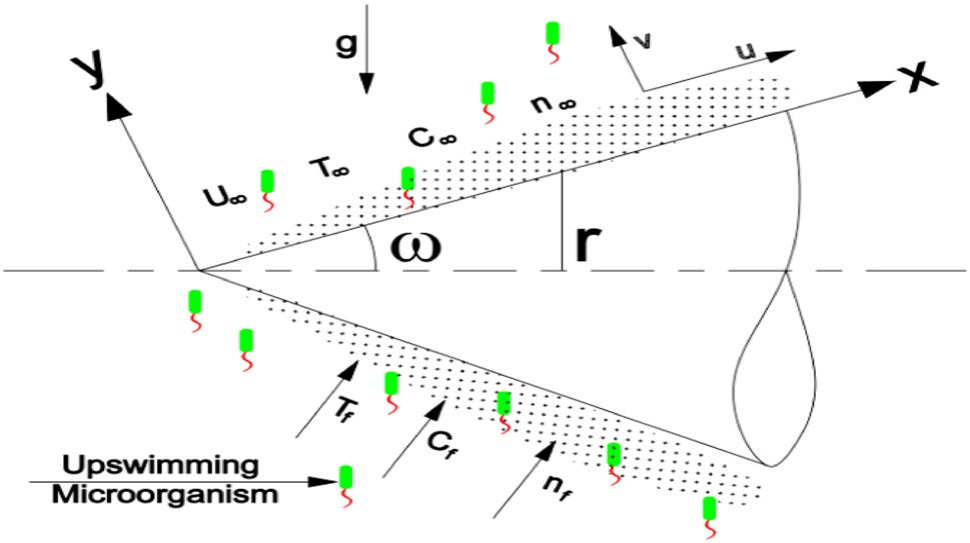
Physical model.

Consider the steady flow of mixed convection boundary layer past a semi-vertically angled horizontal cone immersed in a porous fluid-saturated media with ambient temperature $T_{\infty }$ and concentrations $C_{\infty } ,\;n_{\infty }$. The coordinate x is measured from the cone's tip to the ray's end as well as the longitudinal coordinate y is measured normal to it, see Fig. 1. Convection from a warmed flowing fluid is expected to heat the cone's surface with gyrotactic microorganism at constant temperature $T_{f}$, constant concentrations $C_{f} ,\;n_{f}$ with variable heat, mass and motile microorganism coefficients $h_{f} (x),\;h_{m} (x)$ and $h_{n} (x)$ respectively. The diffusion-thermo and thermo-diffusion effects are also believed to be minimal at low concentrations of the diffusing species. Using the non-Darcy model and Boussinesq approximation, the conservation equations such as continuity, momentum equations (Ref.^41^), thermal energy, mass conservation equations (Ref.^14^) and also the equation of microorganism (Ref.^34^) along with the convective boundary condition (Ref.^31^) are considered as follows

Continuity equation

1 $$\frac{\partial (ru)}{{\partial x}} + \frac{\partial (rv)}{{\partial r}} = 0$$

Momentum equation

2 $$\frac{\partial u}{{\partial y}} + \frac{\partial }{\partial y}\left( {\rho \frac{bK*}{\mu }u^{2} } \right) = - \frac{\partial }{\partial x}\left( {\frac{\rho gK\cos \beta }{\mu }\left[ {\beta_{T} (T - T_{\infty } ) + \beta_{C} (C - C_{\infty } ) + \beta_{n} (n - n_{\infty } )} \right]} \right)$$

Thermal energy equation

3 $$u\frac{\partial T}{{\partial x}} + v\frac{\partial T}{{\partial y}} = \frac{\partial }{\partial y}\left. {\left( {\alpha_{e} \frac{\partial T}{{\partial y}}} \right)} \right)$$

Mass conservation equation

4 $$u\frac{\partial C}{{\partial x}} + v\frac{\partial C}{{\partial y}} = \frac{\partial }{\partial y}\left( {\left. {D_{c} \frac{\partial C}{{\partial y}}} \right)} \right)$$

Conservation equation for microorganism

5 $$u\frac{\partial n}{{\partial x}} + v\frac{\partial n}{{\partial y}} + \frac{{bW_{c} }}{{C_{w} - C_{\infty } }}\left( {\frac{\partial }{\partial y}\left( {n\frac{\partial C}{{\partial y}}} \right)} \right) = \frac{\partial }{\partial y}\left( {\left. {D_{e} \frac{\partial n}{{\partial y}}} \right)} \right)$$

With the boundary conditions are of the form:

6 $$\begin{aligned} v & = 0,\; - k\frac{\partial T}{{\partial y}} = h_{f} (x)(T_{f} - T),\; - D_{m} \frac{\partial C}{{\partial y}} = h_{m} (x)(C_{f} - C), \\ & \;\; - D_{n} \frac{\partial n}{{\partial y}} = h_{n} (x)(n_{f} - n)\;{\text{at}}\;y = 0 \\ \end{aligned}$$

7 $$u \to u_{\infty } ,\;T \to T_{\infty } ,\;C \to C_{\infty } ,\;n \to n_{\infty } \;{\text{at}}\;y \to \infty$$

where T, C, n are temperature, concentration and volume fraction of motile microorganism.

$\alpha_{e} ,\;D_{c} ,\;D_{e}$ are the effective thermal, solutal and microorganism diffusivities and these can be written as according to^14^,

$$\begin{aligned} \alpha_{e} & = \alpha + adu \\ D_{c} & = D_{m} + bdu \\ D_{e} & = D_{n} + cdu \\ \end{aligned}$$

where $\alpha ,\;D_{m} ,\;D_{n}$ are the constant thermal, molecular and microorganism diffusivities and $a,\;b,\;c$ are the coefficients of the thermal, solutal and microorganism dispersions sequentially. And the values of a, b, c lie between $\frac{1}{7}$ and $\frac{1}{3}$.

Introducing the dimensionless quantities listed below

8 $$\begin{aligned} \eta & = \frac{y}{x}Pe_{x}^{\frac{1}{2}} \left( {1 + \frac{{Ra_{x}^{\frac{1}{3}} }}{{Pe_{x}^{\frac{1}{2}} }}} \right)\;\psi = \alpha r\;Pe_{x}^{\frac{1}{2}} \left( {1 + \frac{{Ra_{x}^{\frac{1}{3}} }}{{Pe_{x}^{\frac{1}{2}} }}} \right)f(\eta ), \\ \theta (\eta ) & = \frac{{T - T_{\infty } }}{{T_{f} - T_{\infty } }},\;\;\phi (\eta ) = \frac{{C - C_{\infty } }}{{C_{f} - C_{\infty } }},\;\;\chi (\eta ) = \frac{{n - n_{\infty } }}{{n_{f} - n_{\infty } }} \\ \end{aligned}$$

9 $$h_{f} (x) = x^{{ - \frac{1}{2}}} h_{f} ,\;\;h_{m} (x) = x^{{ - \frac{1}{2}}} h_{m} ,\;\;h_{n} (x) = x^{{ - \frac{1}{2}}} h_{n}$$

where ψ is the stream function, as is customarily defined

$$u = \frac{1}{r}\frac{\partial \psi }{{\partial y}},\;\;v = - \frac{1}{r}\frac{\partial \psi }{{\partial x}}$$

Transformed ordinary differential equations

10 $$f^{\prime\prime} + 2\lambda^{2} {\text{Re}} f^{\prime}f^{\prime\prime} + (1 - \lambda )^{3} \left[ { - \frac{{\eta \theta^{\prime}}}{2} - \frac{{\eta \theta^{\prime}}}{6}(1 - \lambda ) + N_{1} \left( { - \frac{{\eta \phi^{\prime}}}{2}} \right. - \frac{{\eta \phi^{\prime}}}{6}(1 - \lambda ) + N_{2} \left( { - \frac{{\eta \chi^{\prime}}}{2}} \right. - \frac{{\eta \chi^{\prime}}}{6}(1 - \lambda )} \right] = 0$$

11 $$\theta^{\prime\prime} + \frac{1}{2}f\theta^{\prime} - \frac{{f\theta^{\prime}}}{6}(1 - \lambda ) + \lambda^{2} aPe_{d} \left( {f^{\prime}\theta^{\prime\prime} + f^{\prime\prime}\theta^{\prime}} \right) = 0$$

12 $$\phi^{\prime\prime} + \frac{1}{2}Lef\phi^{\prime} - Le\frac{{f\phi^{\prime}}}{6}(1 - \lambda ) + Le\lambda^{2} bPe_{d} (f^{\prime}\phi^{\prime\prime} + f^{\prime\prime}\phi^{\prime}) = 0$$

13 $$\chi^{\prime\prime} + \frac{1}{2}Lbf\chi^{\prime} - Lb\frac{{f\chi^{\prime}}}{6}(1 - \lambda ) + Lb\lambda^{2} cPe_{d} (f^{\prime}\chi^{\prime\prime} + f^{\prime\prime}\chi^{\prime}) - Pe\left[ {\phi^{\prime}\chi^{\prime} + (\chi + \omega )\phi^{\prime\prime}} \right] = 0$$

Boundary condition becomes

14 $$\begin{aligned} f(0) & = 0,\;\theta^{\prime}(0) = - \lambda B_{i} (1 - \theta (0)),\;\phi^{\prime}(0) = - \lambda B_{i,m} (1 - \phi (0)),\;\chi^{\prime}(0) = - \lambda B_{i,n} (1 - \chi (0)) \\ & \;\;{\text{at}}\;\eta = 0 \\ \end{aligned}$$

15 $$f^{\prime}(\infty ) = \lambda^{2} ,\;\theta (\infty ) = = 0,\;\phi (\infty ) = 0,\;\chi (\infty ) = 0\;{\text{at}}\;\eta \to \infty$$

where Local Rayleigh number $Ra_{x} = \frac{{kg\beta_{T} (T_{f} - T_{\infty } )\cos \omega .x}}{\nu \alpha }$, Local peclet number $Pe_{x} = \frac{{u_{\infty } x}}{\alpha }$, Mixed Convective term $\lambda = \frac{1}{{1 + \frac{{Ra_{x}^{\frac{1}{3}} }}{{Pe_{x}^{\frac{1}{2}} }}}}$,Buoyancy Ratio parameter $N_{1} = \frac{{\beta_{C} (C_{f} - C_{\infty } )}}{{\beta_{T} (T_{f} - T_{\infty } )}}$, Buoyancy Ratio parameter $N_{2} = \frac{{\beta_{n} (n_{f} - n_{\infty } )}}{{\beta_{T} (T_{f} - T_{\infty } )}}$, Lewis number $Le = \frac{\alpha }{{D_{m} }}$, Bioconvection Lewis number $Lb = \frac{\alpha }{{D_{n} }}$, Bioconvection peclet number $Pe = \frac{{bW_{c} }}{{D_{n} }},$ Microorganism concentration difference parameter $A = \frac{{n_{\infty } }}{{n_{w} - n_{\infty } }}$, mixed convection parameter $\lambda = \frac{1}{{1 + \frac{{Ra_{x}^{\frac{1}{3}} }}{{Pe_{x}^{\frac{1}{2}} }}}},$ Biot number $B_{i} = - \frac{{h_{f} \alpha^{\frac{1}{2}} }}{{ku_{\infty }^{\frac{1}{2}} }}$.

Biot number of mass transfer $B_{{i,m}} = \frac{{h_{m} \alpha ^{{\frac{1}{2}}} }}{{D_{m} u_{\infty } ^{{\frac{1}{2}}} }},$ Biot number of microorganism transfer $B_{{i,n}} = \frac{{h_{n} \alpha^{\frac{1}{2}} }}{{D_{n} u_{\infty }^{\frac{1}{2}} }},$ Inertia coefficient dependent Reynolds number ${\text{Re}} = \frac{{K^{*} b}}{\nu }u_{\infty }$. The local Nusselt number $Nu_{x} ,$ Sherwood number $Sh_{x}$ and local density number of the motile microorganisms $Nn_{x}$ are expressed as

16 $$Nu_{x} = \frac{{xq_{w} }}{{k (T_{f} - T_{\infty } )}}, \;Sh_{x} = \frac{{xq_{m} }}{{D (C_{f} - C_{\infty } )}},\; Nn_{x} = \frac{{xq_{n} }}{{D_{n} (n_{f} - n_{\infty } )}}$$

where the wall heat, wall mass, and wall motile microorganisms fluxes are specified as $q_{w}$, $q_{m} ,$ and $q_{n}$ respectively and the way they are defined as

17 $$q_{w} = - k\left( {\frac{\partial T}{{\partial y}}} \right)_{y = 0 } ,\;q_{m} = - D_{B} \left( {\frac{\partial C}{{\partial y}}} \right)_{y = 0 } ,\;q_{n} = - D_{n} \left( {\frac{\partial n}{{\partial y}}} \right)_{y = 0 }$$

The dimensionless forms of local nusselt number, local Sherwood number, and local motile microorganism are as follows,

18 $$\lambda Pe_{x}^{{\frac{ - 1}{2}}} Nu_{x} = - \theta^{\prime}\left( 0 \right) , \;\lambda Pe_{x}^{{\frac{ - 1}{2}}} Sh_{x} = - \phi^{\prime}\left( 0 \right),\;\lambda Pe_{x}^{{\frac{ - 1}{2}}} Nn_{x} = - \chi^{\prime}\left( 0 \right)$$

## Numerical method

Simulation of the transformed Eqs. (10–13) within the confines of the boundary conditions (14), (15) are found for various values of the flow regulating parameters using the Matlab BVP4C numerical technique. The governing equations must be transformed into first order differential equations in the context of the bvp4c function stated above. At first Eqs. (10–13) can be rearranged in the following way

$$\begin{aligned} f^{\prime\prime} & = \frac{{ - (1 - \lambda )^{3} \left( {\frac{\lambda }{6} - \frac{2}{3}} \right)\eta \left[ {\theta^{\prime} + N_{1} \phi^{\prime} + N_{2} \chi^{\prime}} \right]}}{{\left( {1 + 2{\text{Re}} f^{\prime}} \right)}} \\ \theta^{\prime\prime} & = - \left( {\frac{\lambda }{6} + \frac{1}{3}} \right)f\theta^{\prime} - \lambda^{2} aPe_{d} \left( {f^{\prime}\theta^{\prime\prime} + f^{\prime\prime}\theta^{\prime}} \right) \\ \phi^{\prime\prime} & = - \left( {\frac{\lambda }{6} + \frac{1}{3}} \right)Lef\phi^{\prime} - Le\lambda^{2} bPe_{d} \left( {f^{\prime}\phi^{\prime\prime} + f^{\prime\prime}\phi^{\prime}} \right) \\ \chi^{\prime\prime} & = - \left( {\frac{\lambda }{6} + \frac{1}{3}} \right)Lbf\chi^{\prime} - Lb\lambda^{2} cPe_{d} \left( {f^{\prime}\chi^{\prime\prime} + f^{\prime\prime}\chi^{\prime}} \right) + Pe\left[ {\phi^{\prime}\chi^{\prime} + (\chi + A)\phi^{\prime\prime}} \right] \\ \end{aligned}$$

This equation must now be transformed into a first order differential equation. For this let $\eta =x$ and

$$y_{1} = f, \;\; y_{2} = f^{\prime}$$

$$y_{3} = \theta ,\;\; y_{4} = \theta^{\prime}, \;\;y_{5} = \phi ,$$

$$y_{6} = \phi^{\prime},\;\; y_{7} = \chi ,\;\; y_{8} = \chi ^{\prime}$$

The first order differential equations are as follows:$$\begin{aligned} \frac{{dy_{1} }}{dx} & = f^{\prime} = y_{2} \\ \frac{{dy_{2} }}{dx} & = f^{\prime\prime} = \frac{{ - (1 - \lambda )^{3} \eta \left( {\frac{\lambda }{6} - \frac{2}{3}} \right)\left[ {y_{4} + N_{1} y_{6} + N_{2} y_{8} } \right]}}{{(1 + 2\lambda^{2} {\text{Re}} y_{2} )}} \\ \frac{{dy_{4} }}{dx} & = \theta^{\prime\prime} = \frac{{ - \left( {\frac{\lambda }{6} + \frac{1}{3}} \right)y_{1} y_{4} - \lambda^{2} aPe_{d} y_{4} \left( { - \frac{{ - (1 - \lambda )^{3} \eta \left( {\frac{\lambda }{6} - \frac{2}{3}} \right)\left( {y_{4} + N_{1} y_{6} + N_{2} y_{8} } \right)}}{{1 + \lambda^{2} {\text{Re}} y_{2} }}} \right)}}{{1 + \lambda^{2} aPe_{d} y_{2} }} \\ \frac{{dy_{6} }}{dx} & = \phi^{\prime\prime} = \frac{{ - Le\left( {\frac{\lambda }{6} + \frac{1}{3}} \right)y_{1} y_{6} - Le\lambda^{2} bPe_{d} y_{6} \left( { - \frac{{ - (1 - \lambda )^{3} \eta (\frac{\lambda }{6} - \frac{2}{3})(y_{4} + N_{1} y_{6} + N_{2} y_{8} )}}{{1 + \lambda^{2} {\text{Re}} y_{2} }}} \right)}}{{1 + Le\lambda^{2} bPe_{d} y_{2} }} \\ \frac{{dy_{8} }}{dx} & = \chi^{\prime\prime} = \frac{\begin{gathered} - Lb\left( {\frac{\lambda }{6} + \frac{1}{3}} \right)y_{1} y_{8} - Lb\lambda^{2} cPe_{d} y_{8} \left( { - \frac{{(1 - \lambda )^{3} \eta (\frac{\lambda }{6} - \frac{2}{3})(y_{4} + N_{1} y_{6} + N_{2} y_{8} )}}{{1 + \lambda^{2} {\text{Re}} y_{2} }}} \right) + Pe(y_{6} y_{8} + \hfill \\ (y_{7} + A)\left( {\frac{{ - Le\left( {\frac{\lambda }{6} + \frac{1}{3}} \right)y_{1} y_{6} - Le\lambda^{2} bPe_{d} y_{6} ( - \frac{{ - (1 - \lambda )^{3} \eta \left( {\frac{\lambda }{6} - \frac{2}{3}} \right)(y_{4} + N_{1} y_{6} + N_{2} y_{8} )}}{{1 + \lambda^{2} {\text{Re}} y_{2} }}}}{{1 + Le\lambda^{2} bPe_{d} y_{2} }}} \right) \hfill \\ \end{gathered} }{{1 + Lb\lambda^{2} cPe_{d} y_{2} }} \\ \end{aligned}$$.

The boundary conditions become considering $ya$ be the left boundary, $yb$ be the right boundary

$$\begin{aligned} & ya(1) = 0,\;yb(2) - \lambda ^{2} = 0 \\ & ya(4) + \lambda B_{i} (1 - ya(3)) = 0,\;yb(3) = 0 \\ & ya(6) + \lambda B_{{i,m}} (1 - ya(5)) = 0,\;yb(5) = 0 \\ & ya(8) + \lambda B_{{i,n}} (1 - ya(7)) = 0,\;yb(7) = 0 \\ \end{aligned}$$

The MATLAB algorithm's accuracy and dependability have been proven in a number of recent researches papers. Table 1 shows a clear comparison of the current findings with those of Reddy^22^ for a few special circumstances, and there is a strong correlation.

**Table 1:** Comparison of $f^{\prime}(0),\; - \theta^{\prime}(0),\; - \phi ^{\prime}(0)$ for $\lambda = 1,\;{\text{Re}} = 0,\;Pe_{d} = 0,\;Lb = 0,\;Pe = 0,\;B_{i} \to \infty ,\;B_{i,m} \to \infty$.

		When $Le = 1$				When *Le* = 10			
$f^{\prime}(0)$		$- \theta^{\prime}(0)$		$- \phi ^{\prime}(0)$		$- \theta^{\prime}(0)$		$- \ \phi ^{\prime}(0)$	
Reddy^22^	Present result	Reddy^22^	Present result	Reddy^22^	Present result	Reddy^22^	Present result	Reddy^22^	Present result
1.0000	1.0000	0.5642	0.5644	0.5642	0.5644	0.5642	0.5644	1.7841	1.7841

## Result discussion

**Figure 2:**
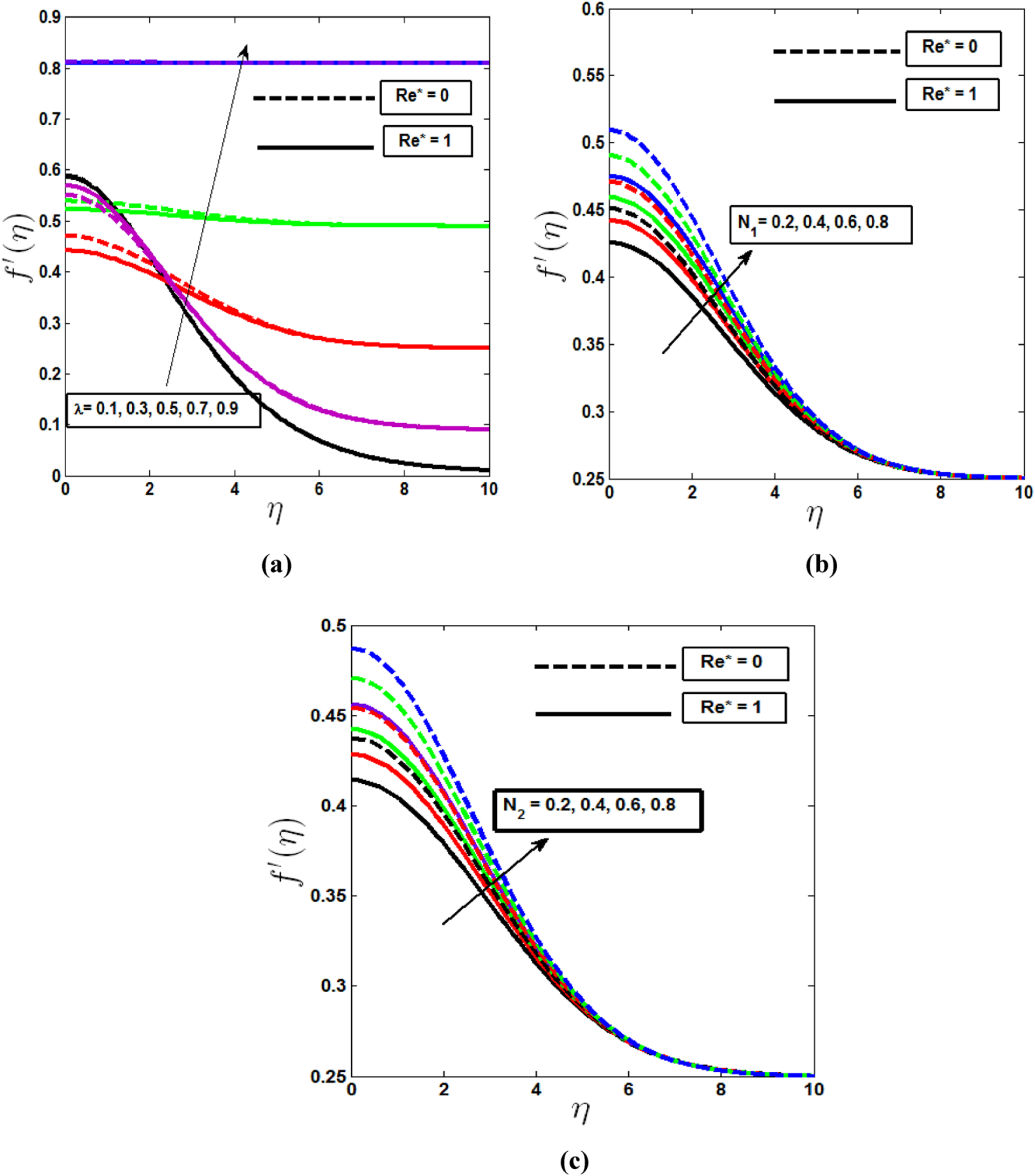
Velocity profile with the variation of (**a**) mixed convection parameter λ, (**b**) Buoyancy parameter $N_{1}$, (**c**) Buoyancy parameter $N_{2}$ for Darcy and non-Darcy cases.

In Fig. 2 the effect of mixed convection and buoyancy parameters on the velocity profile is demonstrated. With rising λ values, the velocity profile increases in Fig. 2a which indicates velocity profile gradually increases for free to forced convection for the inciting attitude of buoyancy forces for up swimming microorganism. Fig. 2b, c depicts that increase in buoyancy parameters $N_{1} ,N_{2}$ resulting in an increase in the velocity profile. Because the buoyancy parameter is proportional to the buoyancy, the larger the buoyancy parameter, the greater the buoyancy. These higher levels of buoyancy operate as agents, increasing fluid velocity. And in every case it is observed velocity profile mostly increases for Darcy porous media comparing to non-Darcy case.

**Figure 3:**
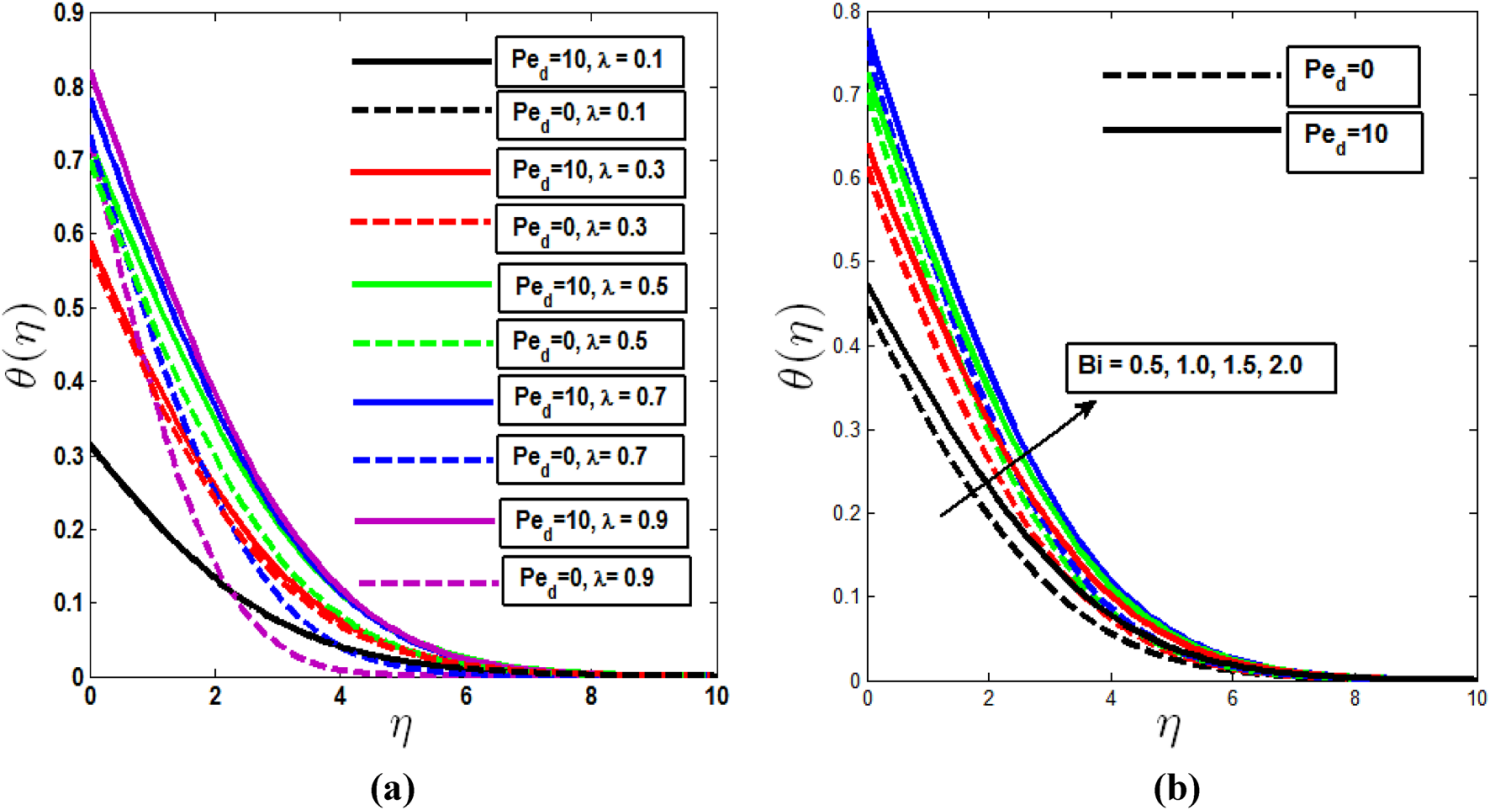
Temperature profile with the (**a**) variation of mixed convection parameter, (**b**) Biot number in the presence and absence of thermal dispersion effect.

Effect of temperature profile with the growing values of mixed convection parameter λ and Biot number $B_{i}$ are observed in Fig. 3. Fig. 3a depicts temperature profile increases which mixed convection parameter λ in the presence of dispersion effect because of the increment of motion of the fluid average kinetic energy increases which causes increment of temperature profile. But it is seen when dispersion effect is absent temperature profile increases from free to mixed convection regime, then again decreases to forced convection regime. In Fig. 3b As the Biot number rises, the temperature profile rises with it. The Biot number helps to increase the temperature profiles of the fluid by increasing internal heat in solid surfaces. When there is a dispersion effect, the thickness of the boundary layer of the temperature profile increases.

**Figure 4:**
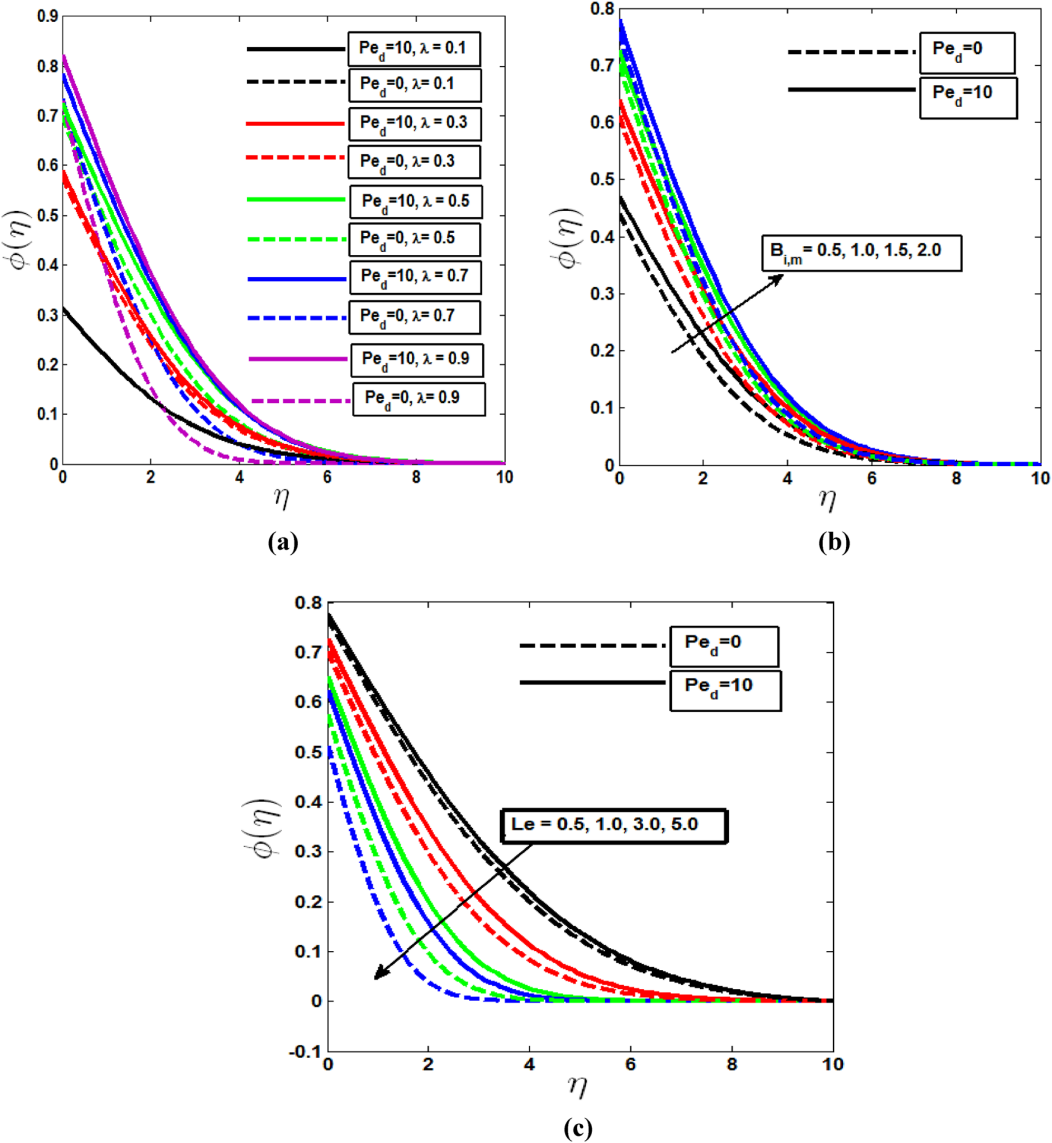
Concentration profile with the (**a**) variation of mixed convection parameter, (**b**) Biot number of mass transfer, (**c**) Lewis parameter Le in the presence and absence of solutal dispersion effect.

**Figure 5:**
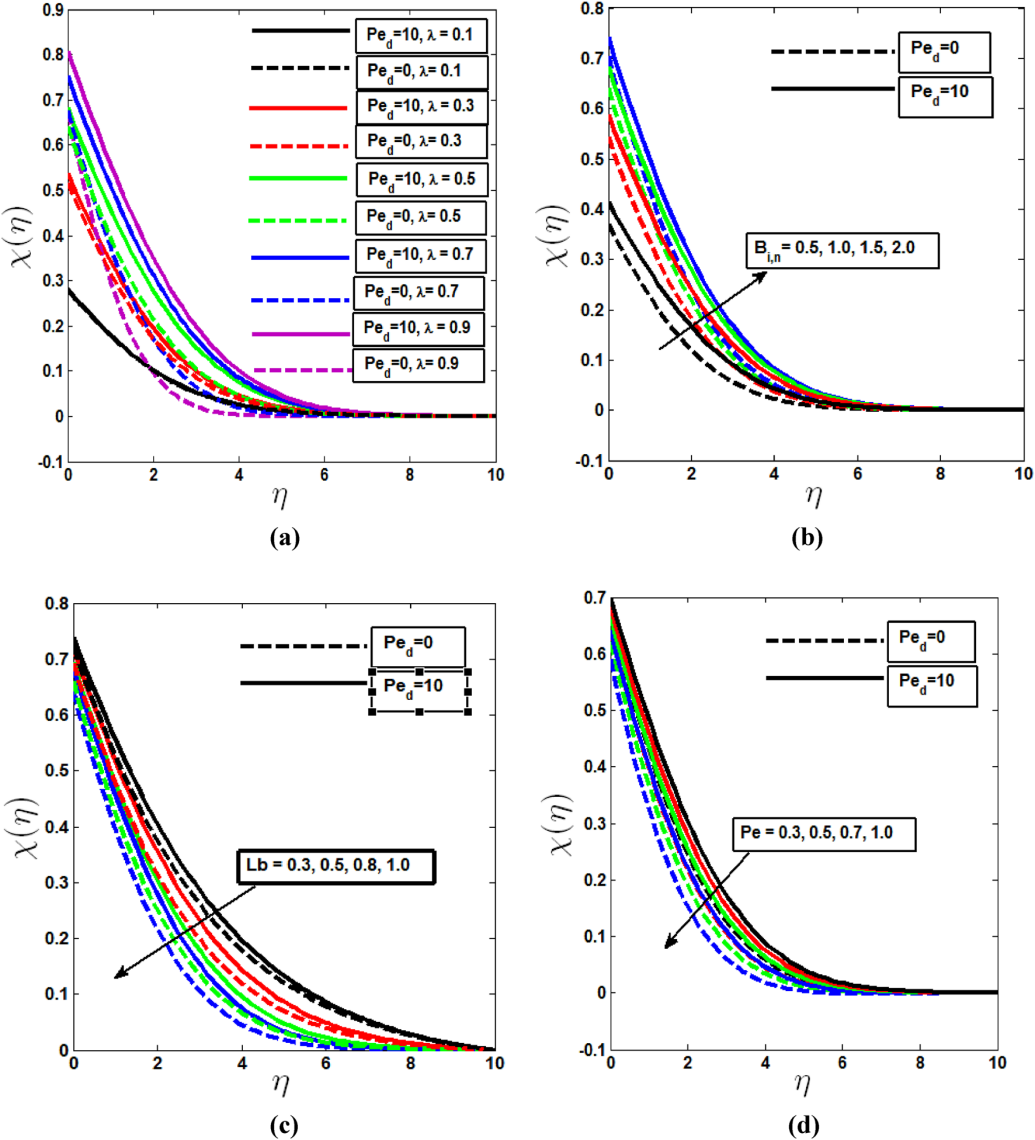
Microorganism profile with the (**a**) variation of mixed convection parameter, (**b**) Biot number of microorganism transfer, (**c**) Bioconvection Lewis parameter Le, (**d**) Bioconvection peclet number in the presence and absence of microorganism dispersion effect.

The impact of the mixed convection parameter on concentration and microorganism profile is demonstrated in Figs. 4a and 5a. In both cases boundary layer thickness are increasing with λ in the presence of dispersion effect. Without dispersion effect both profiles increases to pure free convection (λ = 0) to pure mixed convection (λ = 0.5) and then decreases to pure forced convection (λ = 1). Figures 4b and 5b depict that the concentration and microorganism profiles increase within the boundary layer when Biot number of mass transfer $B_{i,m}$ and Biot number of motile microorganism transfer $B_{i,n}$ increase from least to large value. Biot numbers are placed as a boundary condition in the enhanced wall boundary condition in Eq. (13). More heat and species of motile microorganisms are conveyed to the fluid as Biot Numbers rise, energizing the temperature, concentration, and microorganism profile boundary layer.

The ratio of thermal diffusivity to mass (Nano-particle) species diffusivity is known as the Lewis number. Le = 1 denotes that the fluid's thermal diffusivity and species diffusivity are the same, as well as the thickness of both boundary layers. When Le is less than one, mass diffusivity is greater than thermal diffusivity, and vice versa when Le is greater. In Fig. 4c the mass diffusivity of the concentration boundary layer decreases as the Lewis number decreases, lowering the penetration depth. And without the dispersion effect, the concentration profile deteriorates. Bioconvection Peclet number Pe and the bioconvection Lewis number Lb have a propensity to lower the motile microorganism density. Bioconvection peclet number Pe and The quantity of motile microbe thickness decreases as the Lewis number Lb of bioconvection increases fluid mobility which are shown in Fig. 5c and d. The behavior of Microorganism profile with and without dispersion effects are also shown in Fig. 5.

**Figure 6:**
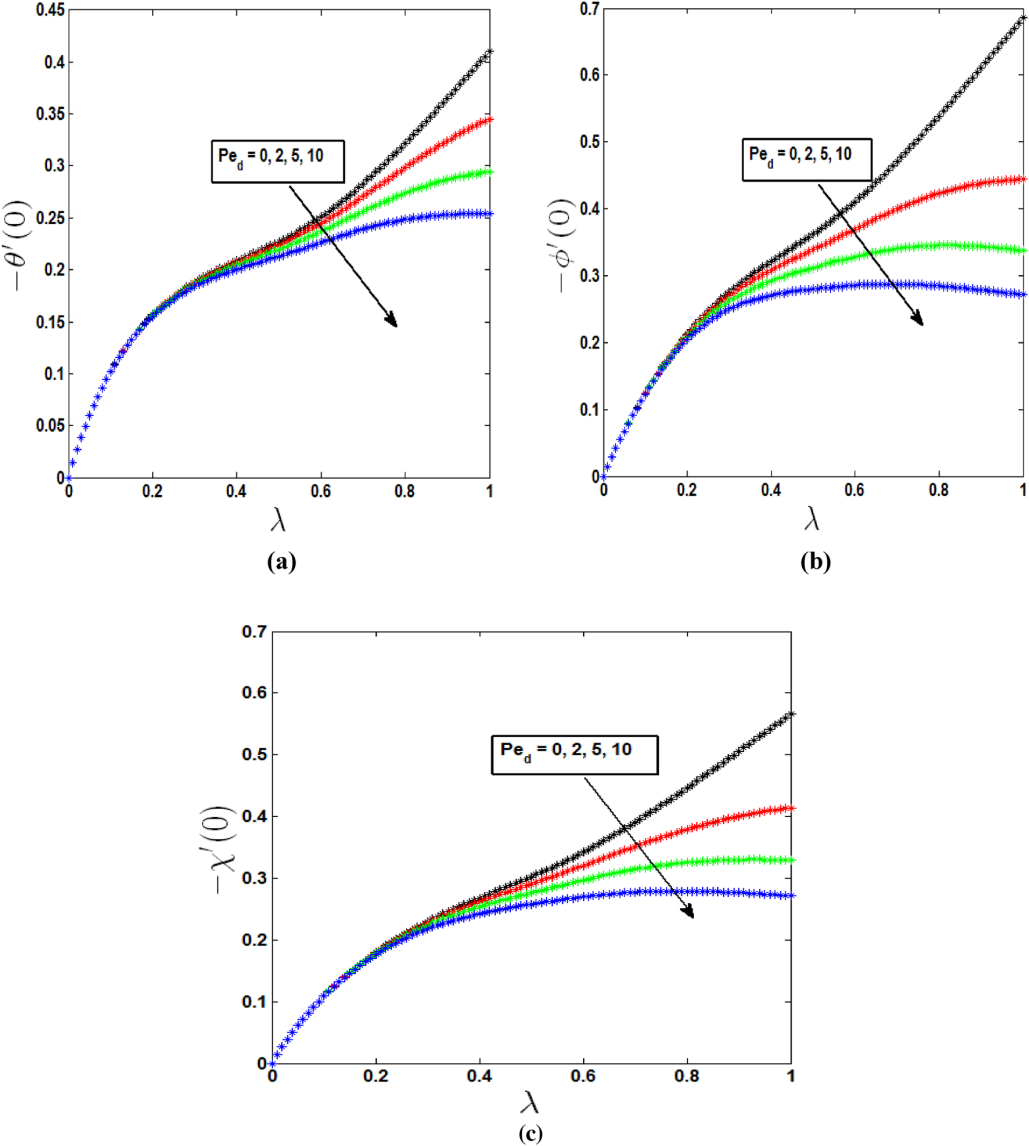
Dispersion effects on (**a**) heat transfer rate, (**b**) mass transfer rate, (**c**) motile microorganism transfer rate in free, forced and mixed convection regime.

Figure 6 shows the impacts of dispersion on heat, mass, and motile microbe transfer rates as the mixed convection parameter is varied. The rate of heat transmission increases as the dispersion parameter decreases. In Fig. 6a it is observed that heat transfer rate is higher for the absence of dispersion effect. As we know λ is closer to 0 indicates free convection regime and closer to 1 indicates forced convection regime, in Fig. 6a it is noticeable that dispersion effect on heat transfer rate is negligible in free convection region. On the other hand effects are prominent in forced convection region. Similarly Fig. 6b and c depict mass and motile microorganism transfer rate are increasing with the growing values of λ when there is negligible dispersion effect. When c = 0.3 and $Pe_{d} = 5$ mass and motile microorganism transfer rate increase from λ = 0 to λ = 0.82 and also when c = 0.3 and $Pe_{d} = 10$ mass and motile microorganism transfer rate increase from λ = 0 to λ = 0.69, after that in both cases mass and microorganism transfer rate gradually decrease. So decreasing phenomena is observed in forced convective region in the presence of dispersion effect.

**Figure 7:**
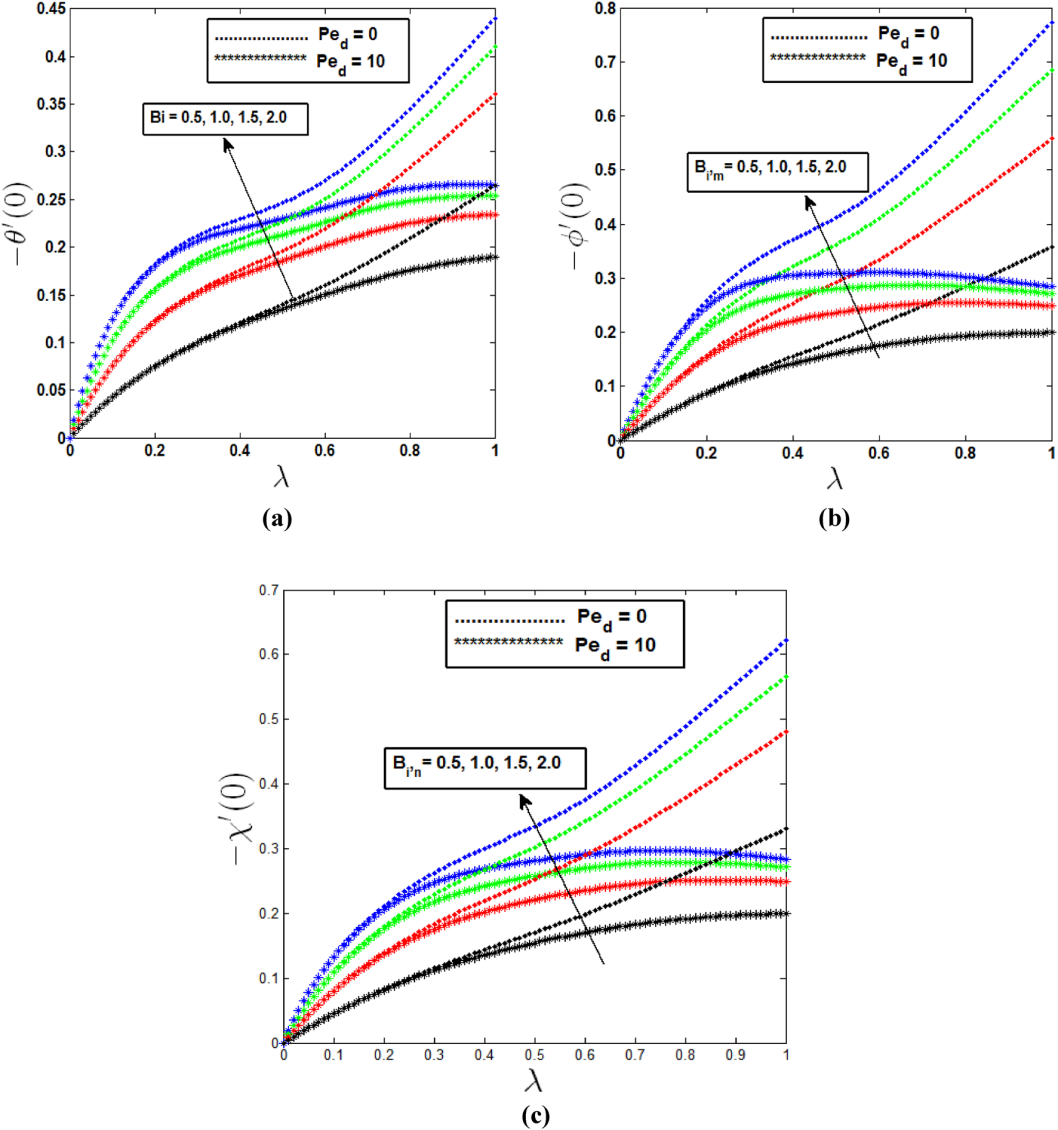
Effects of Biot numbers on (**a**) heat transfer rate, (**b**) mass transfer rate, (**c**) Motile microorganism transfer rate in free, forced and mixed convection regime.

For the growing effects of Biot numbers, heat, mass, and microbe transfer rates are observed in Fig. 7 as the mixed convection parameter is varied. An increase in the Biot number brings an increase in the fluid temperature Due to convective heat transfer from the hot fluid to the surface of the cone, the surface gets heated which in turn increases the heat transfer rate from the surface to the fluid. So In Fig. 7 we can see heat, mass and motile microorganism transfer rates are increasing from λ = 0 to λ = 1 when dispersion effect is absent. But this increasing rate is not similar in all regions. In the mixed convection region it is seen in Fig. 7a–c that increasing phenomena is comparatively slower than free and forced convective region. On the other hand when dispersion effects are present heat transfer rate decreases after when $B_{i} = 1.5$ for $\lambda = 0.98$ and $B_{i} = 2$ for $\lambda = 0.94$ which is closer to pure forced convection regime. And also mass transfer rate decreases after when $B_{i,m} = 1$ for $\lambda = 0.79$ $B_{i,m} = 1.5$ for $\lambda = 0.69$ and $B_{i,m} = 2$ for $\lambda = 0.62$.Similarly when $B_{i,n} = 1$ for $\lambda = 0.86$ $B_{i,n} = 1.5$ for $\lambda = 0.80$ and $B_{i,n} = 2$ for $\lambda = 0.74$, motile microorganism transfer rate drops.

**Figure 8:**
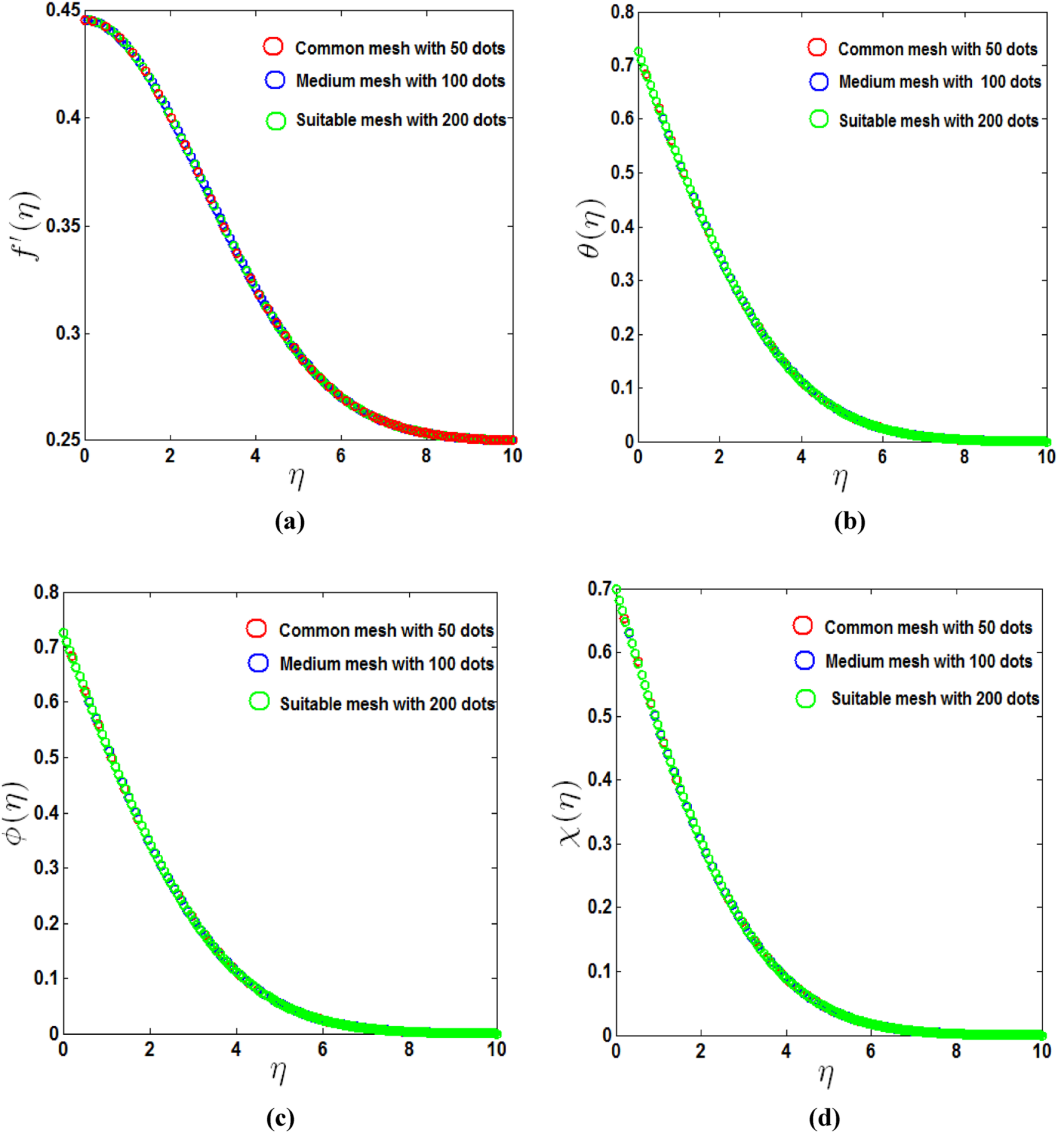
Grid Independence test (**a**) velocity profile $f^{\prime}(\eta )$, (**b**) temperature profile $\theta (\eta )$, (**c**) concentration profile $\phi (\eta )$, (**d**) microorganism profile $\chi (\eta )$.

Figure 8 depicts the grid-independence test. The grid convergence test is used to maintain the point of exactness. It started with a common mesh with 50 points. We get the medium mesh, 100 points of accuracy, and suitable mesh, 200 points of accuracy for velocity, temperature, concentration and microorganism profiles by increasing the number of points twice and triple. When the number of points exceeds the suitable mesh number of points, the precision is unaffected, but the set time is increased.

## Conclusion

Theoretical and numerical studies are conducted on a new mathematical model for stable two-dimensional mixed convection flow via a horizontal cone containing gyrotactic microorganisms with convective boundary conditions. The influences of dispersion effects on the rate of transport of heat, mass, and motile microorganisms along with velocity, temperature, concentration and microorganism profiles are observed. The key findings of this analysis can be summarized as follows:

- As the value of the mixed convection parameter λ rises, the velocity profile and buoyancy parameters $N_{1} ,N_{2}$ rise as well especially for Darcy porous media.
- Temperature, Concentration and Microorganism profiles are increasing with increasing values of λ when there is no dispersion effect. But in the presence of dispersion effect the profiles increase for free (λ = 0) to pure mixed convection (λ = 0.5), then decrease for those values of λ which indicate forced convection.
- Dispersion effect has great influence on the rate of heat, mass, and motile microbe transfer. For higher effects of dispersion the rate of heat, mass and motile microorganism transfer increase in a free-convection zone and drops in forced convection region.
- The Biot number increases the rate of heat, mass and motile microorganism transfer from free to forced convection, with no influence on dispersion. The flow transfer rates decrease with the influence of the effect of dispersion.

## Data Availability

The data that support the results of this research work are available from the corresponding author upon the request.
